# Distinct contributions of cerebrospinal fluid biomarkers to cognitive impairment and neuropsychiatric symptoms in young-onset dementia

**DOI:** 10.1017/neu.2025.10051

**Published:** 2025-12-23

**Authors:** Wei-Hsuan Chiu, Anita M.Y. Goh, Dhamidhu Eratne, Charles B. Malpas, Matthew Jee Yun Kang, Wendy Kelso, Mark Walterfang, Dennis Velakoulis, Samantha M. Loi

**Affiliations:** 1 Department of Psychiatry, The University of Melbourne, Parkville, VIC, Australia; 2 Neuropsychiatry Centre, The Royal Melbourne Hospital, Parkville, VIC, Australia; 3 National Ageing Research Institute, Parkville, VIC, Australia; 4 The University of Melbourne, Parkville, VIC, Australia; 5 The Royal Melbourne Hospital, Parkville, VIC, Australia; 6 Department of Neurology, The Royal Melbourne Hospital, Parkville, VIC, Australia; 7 Department of Medicine (Royal Melbourne Hospital), The University of Melbourne, Parkville, VIC, Australia; 8 Melbourne School of Psychological Sciences, The University of Melbourne, Parkville, VIC, Australia; 9 Florey Institute of Neuroscience and Mental Health, Parkville, VIC, Australia; 10 Centre for Ageing and Alzheimer’s Disease, School of Medicine and Health Sciences, Edith Cowan University, Joondalup, WA, Australia

**Keywords:** Early onset Alzheimer disease, cognitive dysfunction, affective symptoms, biomarkers, neurofilament protein L

## Abstract

**Objective:**

Young-onset dementia (YOD), defined by symptom onset before age 65, encompasses diverse aetiologies and presents with prominent neuropsychiatric symptoms (NPS) that often accompany or exacerbate cognitive decline. However, the pathological mechanisms linking NPS, cognition, and biomarkers remain unclear. It was hypothesised that relationships between NPS and cognition would be mediated or moderated by cerebrospinal fluid (CSF) biomarker levels in individuals with YOD.

**Methods:**

This retrospective, cross-sectional study included 46 participants with YOD (24 with Alzheimer’s disease [AD], 22 with non-AD dementias) diagnosed at the Neuropsychiatry Centre, Royal Melbourne Hospital. NPS were measured using the Depression Anxiety and Stress Scale and Cambridge Behavioural Inventory-Revised. Cognition was assessed using standardised neuropsychological assessments. CSF amyloid-β (Aβ42), phosphorylated tau 181 (P-tau181), total tau (T-tau), and neurofilament light chain protein (NfL) were analysed. General linear models (GLMs) examined associations between biomarkers, cognition, and NPS.

**Results:**

Higher P-tau181 (unstandardised beta [B] = −0.10, 95% confidence interval = [−0.20, −0.01]) and T-tau (B = −0.06 [−0.13, −0.01]) levels were associated with poorer memory recall in participants with YOD. In non-AD dementias, higher T-tau levels predicted greater NPS severity (B = 0.76 [0.06, 3.52]). NfL showed no significant associations with NPS or cognition.

**Conclusion:**

Tau-related neurodegeneration (P-tau181 and T-tau) appears more closely linked to memory impairment in YOD than axonal injury markers such as NfL. In non-AD dementias, T-tau was additionally associated with behavioural symptom severity, suggesting tau-related mechanisms across subtypes. These associations require validation in larger, longitudinal, and multimodal studies to clarify temporal and mechanistic pathways.

Significant outcomes

- P-tau181 and T-tau levels were significantly associated with memory recall impairment across young-onset dementia (YOD) groups, highlighting tau-mediated neurodegeneration as a key correlate.
- In non-AD dementias, higher T-tau levels were uniquely linked to greater neuropsychiatric symptom (NPS) severity, suggesting a distinctive behavioural pathology associated with tau.
- Anxiety and stress symptoms were associated with impaired recognition memory independently of core dementia biomarkers, indicating potential non-degenerative mechanisms affecting cognition.

Limitations

- The inclusion of a diagnostically heterogeneous cohort, including various non-AD dementia subtypes, may have introduced variability in pathological mechanisms that were not fully accounted for.
- The small sample size, especially within subgroups, reduced statistical power and may limit the generalisability of the findings.
- The cross-sectional design limited the ability to examine causal or temporal relationships between biomarkers, cognition, and NPS.

## Introduction

Young-onset dementia (YOD) is defined as dementia with onset of the first symptom before the age of 65 (Rossor *et al*., 2010). Although less common than older-onset dementia (onset ≥ 65 years), YOD encompasses a broad spectrum of aetiologies, including but not limited to young-onset Alzheimer’s disease (YOAD), frontotemporal dementia (FTD), vascular dementia (VaD), Huntington’s disease, Parkinson’s disease, and traumatic brain injury (Harvey *et al*., 2003; Ikejima *et al*., 2009; Withall *et al*., 2014; Loi *et al*., 2021). This aetiological diversity contributes to substantial clinical heterogeneity, with symptoms that often differ in their onset, presentation, and progression compared to older-onset cases (Loi *et al*., 2023).

Neuropsychiatric symptoms (NPS), including depression, anxiety, apathy, irritability, and agitation, affect up to 90% of individuals with YOD during the disease course (Mulders *et al*., 2016; Eikelboom *et al*., 2021). These symptoms may precede, accompany, or follow cognitive decline (van Vliet *et al*., 2012; Bauhuis *et al*., 2020), and they substantially worsen patient outcomes and caregiver burden (Bakker *et al*., 2013; Kang *et al*., 2024a). However, NPS are not unique to dementia; they are also observed in cognitively intact individuals and in primary psychiatric disorders (Gatchel *et al*., 2017; Ng *et al*., 2021). Distinguishing NPS arising from neurodegenerative processes versus those of psychiatric or reactive origin remains a major diagnostic and conceptual challenge, particularly in younger adults, where symptom profiles may be atypical (Woolley *et al*., 2011; Tsoukra *et al*., 2022). This distinction is clinically significant, as similar neuropsychiatric presentations in cognitively intact individuals may not reflect underlying neurodegeneration.

Emerging evidence suggests that NPS may not simply co-occur with cognitive impairment but may also contribute to its trajectory. A recent meta-analysis involving 26893 individuals with various dementia types (both young- and older-onset dementia) reported that higher NPS severity was associated with poor performance across multiple cognitive domains, including attention, executive function, memory, and semantic knowledge (Sabates *et al*., 2024). In mild cognitive impairment (MCI), conceptualised as a prodromal stage of dementia, NPS have similarly been linked to accelerated cognitive decline and an increased risk of progression to dementia (Somme *et al*., 2013; Lo *et al*., 2020; McGirr *et al*., 2022). These findings support an interdependent relationship between neuropsychiatric and cognitive symptoms across the dementia spectrum.

Despite their clinical relevance, the pathophysiological mechanisms underlying NPS in dementia remain poorly understood. Much of the research to date has centred on three core Alzheimer’s disease (AD) biomarkers: amyloid-β (Aβ), phosphorylated tau (P-tau), and total tau (T-tau), which form the basis of the ATN (Amyloid, Tau, Neurodegeneration) classification framework (Jack Jr. et al. 2018, 2024). However, the relationship between these biomarkers and NPS remains equivocal. Three systematic reviews have highlighted the mixed and sometimes contradictory findings across studies (Banning *et al*., 2019; Showraki *et al*., 2019; Ng *et al*., 2021). For example, Showraki *et al*. (2019) found that agitation was consistently associated with ATN markers in both MCI and AD cohorts, while Banning *et al*. (2019) reported no association between agitation and tau or neurodegeneration (T and N) markers. These discrepancies may reflect methodological differences, variability in symptom definitions, and the influence of disease stage, but also point to an intricate interplay between cognition and NPS (Gatchel et al. 2019; Ng *et al*., 2021). Furthermore, emerging evidence suggests that Aβ may act as a moderator in the relationship between affective symptoms and cognitive decline (Johansson *et al*., 2020).

In addition to ATN biomarkers, neurofilament light chain protein (NfL), a marker of axonal injury, has gained attention as a sensitive, non-specific indicator of neurodegeneration across diverse conditions, including AD, FTD, and VaD (Meeter *et al*., 2017; Khalil *et al*., 2018, 2024; Zhao *et al*., 2019). Unlike T-tau, which is more specific to AD, NfL captures broader neuroaxonal damage and may therefore be particularly useful in heterogeneous clinical populations such as YOD (Alirezaei *et al*., 2020; Eratne et al. 2022, 2024a, 2024b; Zetterberg and Bendlin, 2021). Elevated NfL has been associated with a range of NPS, including psychosis, depression, anxiety, apathy, disinhibition, sleep disturbance, and aberrant motor behaviour, in both MCI and dementia cohorts (Bloniecki *et al*., 2020; Gomar and Koppel, 2024; Huang *et al*., 2024). Moreover, NfL may bridge the gap between NPS and cognitive decline, as suggested by a recent study reporting that NfL mediates the relationship between depressive symptoms and cognitive decline in cognitively normal older adults (Xu *et al*., 2024).

While most of these findings have primarily been derived from older-onset dementia populations, it is unclear whether similar relationships between NPS, cognition, and biomarkers are replicated in younger people with dementia. YOD presents unique challenges due to its earlier onset, psychosocial implications, and complex pathophysiology. The broad range of aetiologies and distinct neurobiological underpinnings of YOD complicate the direct application of findings from older-onset populations (Loi *et al*., 2023). Importantly, no prior cohort studies have systematically examined the interplay between NPS, cognition, and biomarkers in YOD, representing a critical gap in our understanding of these processes.

Therefore, this study aimed to: (1) examine the associations between amyloid-β42, phosphorylated tau181, total tau, and neurofilament light chain protein and both neuropsychiatric symptoms and cognitive functions in individuals with YOD, and (2) identify whether these biomarkers act as potential moderators or mediators in the relationships between neuropsychiatric symptoms and cognition. We hypothesised that (a) CSF T-tau and NfL would show distinct associations with neuropsychiatric and cognitive outcomes, reflecting different neurodegenerative mechanisms, and (b) biomarker levels may moderate or mediate the relationship between NPS and cognition in YOD.

## Material and methods

### Study Design and Participants

This retrospective, cross-sectional study examined the relationships between NPS, cognitive performance, and cerebrospinal fluid (CSF) biomarkers in individuals with YOD. Data were collected from patients diagnosed with YOD at the Neuropsychiatry Centre, The Royal Melbourne Hospital, Victoria, Australia, between April 2009 and December 2021.

Inclusion criteria were: (1) a clinical diagnosis of dementia based on established diagnostic criteria, (2) symptom onset before the age of 65, and (3) completion of neuropsychiatric and cognitive assessments within 12 months of CSF collection. Exclusion criteria included a diagnosis of MCI, primary psychiatric disorders, or secondary causes of cognitive impairment (e.g., metabolic, infectious, or toxic aetiologies).

All patients were assessed by a multidisciplinary team employing multimodal diagnostic approaches (Eratne *et al*., 2020; Loi *et al*., 2022; Kang *et al.,* 2024b). Diagnoses were determined through consensus, with neurodegenerative conditions classified based on established criteria (Armstrong et al. 2013; Emre *et al*., 2007; Bang *et al*., 2015; Jack Jr. et al. 2018; Van Straaten *et al*., 2003; Rascovsky *et al*., 2011; Reilmann *et al*., 2014).

This study was conducted in accordance with the Declaration of Helsinki and was approved by the Human Research Ethics Committee of the Royal Melbourne Hospital and Melbourne Health (approval no. MH 2018.371).

### Cognitive Variables

Cognitive performance was assessed using formal, standardised neuropsychological assessments conducted by clinical neuropsychologists at the Neuropsychiatry Centre as part of a comprehensive diagnostic work-up. Raw data were extracted from clinical files and converted into Z-scores using published normative data, with preference given to Australian norms where available. Cognitive tests were grouped into six primary domains: memory recall, delayed recognition memory, executive function, language, attention and processing speed, and visuospatial function (see Supplementary Table S1 for test details). These domain classifications were informed by published literature (Griffith et al. 2022) and the clinical expertise of neuropsychologists involved in both the assessment and study design (AG, WK, and CM). Mean Z-scores were calculated for each domain to reflect domain-level cognitive functioning.

### Neuropsychiatric Variables

NPS were assessed using two validated tools: the 21-item Depression Anxiety and Stress Scale (DASS-21) (Lovibond and Lovibond, 1995) and the revised Cambridge Behavioural Inventory (CBI-R) (Wear *et al*., 2008). The DASS-21 is a self-report questionnaire measuring depression, anxiety, and stress across three subscales (0–42). Higher scores indicate greater symptom severity, and total scores reflect the overall psychological distress (Lovibond and Lovibond, 1995). The CBI-R is an informant-completed tool measuring the frequency of behavioural symptoms across ten dimensions (*Memory and Orientation*, *Everyday Skills*, *Self-Care*, *Abnormal Behaviour*, *Mood*, *Beliefs*, *Eating Habits*, *Sleep*, *Stereotypic and Motor Behaviours*, and *Motivation*). Higher scores indicate greater symptom severity, and total scores reflect the overall behavioural and functional deficits (Wear *et al*., 2008).

### Biological Variables

CSF samples were collected via lumbar puncture during hospital admissions as part of the routine diagnostic workup. Levels of Aβ42, P-tau181, T-tau, and NfL were measured at the National Dementia Diagnostic Laboratory, Florey Institute of Neuroscience and Mental Health, Melbourne.

Biomarker analyses were conducted following standardised protocols described previously in detail (Eratne *et al*., 2020, 2022; Li et al. 2015). All CSF samples were analysed in duplicate using enzyme-linked immunosorbent assay (ELISA): INNOTEST β-AMYLOID(1−42) (Aβ42), INNOTEST hTAU Ag (T-tau), and INNOTEST PHOSPHO-TAU(181P) (P-tau181) (Innogenetics, Ghent, Belgium), performed as part of clinical biochemical and cytological testing (Li et al. 2015). Residual samples were stored at −80°C for potential reanalysis. CSF NfL concentrations were measured in duplicate using a commercial ELISA (NF-light; UmanDiagnostics, Umea, Sweden) (Eratne *et al*., 2020).

### Statistical Analysis

All statistical analyses were conducted using R version 4.4.1 (R Foundation for Statistical Computing, Vienna, Austria) (R Core Team, 2016). Continuous variables were assessed for normality and log-transformed where necessary to approximate normal distributions. Results from both raw and transformed data were compared for consistency, with log-transformed results reported.

Relationships between NPS, cognitive domains, and biomarkers were assessed using Spearman’s correlation and general linear models (GLMs) with bootstrapping (2000 replicates) to calculate 95% confidence intervals (CIs). This approach mitigates the influence of outliers and non- Gaussian (non-normal) distributions. Statistical significance was defined as *p*-values <0.05 or 95% CIs excluding zero. Descriptive statistics are presented as means (standard deviations [SDs]) for continuous variables and frequencies (percentages) for categorical variables.

#### Primary Analyses

Two primary analytical approaches were undertaken to address the study aims:

**Aim (1)**: GLMs were used to examine associations between biomarkers (independent variables [IVs]) and NPS scores or cognitive domain Z-scores (dependent variables [DVs]). Age was entered as covariate, and sex and diagnosis were included as cofactors in each model (Model 1). Sensitivity analyses included unrelated biomarkers as covariates to assess the independence of observed associations. The Akaike information criterion corrected for small sample sizes (AICc) was applied to measure model fit. The AICc weight indicated the proportion of predictive power provided by the statistical model.

**Aim (2)**: GLMs were used to examine relationships between NPS (IVs) and cognitive domain Z-scores (DVs), adjusting for age, sex, and diagnosis. For significant associations, potential moderating or mediating roles of biomarkers were assessed using GLMs and the PROCESS Macro for R (Model 1: moderation; Model 4: mediation) (Hayes, 2017).

#### Subgroup Analyses

Subgroup analyses were conducted to compare results across the YOD cohort and specific diagnostic subgroups.

#### Correction for Multiple Comparisons

To account for multiple comparisons, the false discovery rate (FDR) correction (Benjamini and Hochberg, 1995) was applied to the GLMs. Adjusted *p*-values are provided in Supplementary Table S2, while uncorrected results are presented in the main text to maintain sensitivity in this exploratory study.

## Results

### Demographics and Clinical Characteristics

This study included 46 participants diagnosed with YOD (33 [72%] males, 13 [28%] females). Among these, 24 were diagnosed with YOAD, 11 with behavioural variant FTD, 3 with other forms of FTD, 2 with VaD, 2 with Parkinson’s disease, 1 with Huntington’s disease, 1 with corticobasal degeneration, 1 with progressive supranuclear palsy, and 1 with central nervous system vasculitis. For analysis, diagnoses were grouped into two categories: YOAD (*n* = 24, 52%) and non-AD dementias (*n* = 22, 48%). Demographic and clinical characteristics of each subgroup are presented in Table 1.

**Table 1.** Study cohort characteristics

Variable		YOAD				Non-AD dementias			
		N	(%)^a^	Mean	(SD)	N	(%)^b^	Mean	(SD)
Total		24				22			
Sex	Male	14	(58)			19	(86)		
Age	Symptom onset (years)	24	(100)	55.3	(6.3)	22	(100)	53.4	(8.8)
	Diagnosis (years)	24	(100)	57.6	(6.3)	22	(100)	56.3	(8.7)
Illness duration	(years)	24	(100)	2.4	(2.3)	22	(100)	2.4	(2.2)
Education	(years)	22	(92)	12.1	(2.7)	19	(86)	12.0	(2.4)
DASS-21	Depression (/42)	22	(92)	8.1	(8.9)	15	(68)	9.9	(6.7)
	Anxiety (/42)	22	(92)	4.5	(4.1)	15	(68)	7.6	(7.1)
	Stress (/42)	22	(92)	8.3	(7.4)	15	(68)	10.5	(7.9)
	Total (/126)	22	(92)	19.2	(19.0)	15	(68)	19.1	(20.2)
CBI-R	Memory and Orientation (/32)	17	(71)	18.0	(8.1)	14	(64)	13.9	(8.7)
	Everyday Skills (/20)	17	(71)	7.4	(6.7)	14	(64)	5.2	(6.0)
	Self-Care (/16)	17	(71)	2.3	(4.7)	14	(64)	2.8	(3.6)
	Abnormal Behaviour (/24)	17	(71)	6.1	(5.0)	14	(64)	5.2	(5.4)
	Mood (/16)	17	(71)	4.6	(3.2)	14	(64)	4.0	(4.7)
	Beliefs (/12)	17	(71)	0.1	(0.3)	14	(64)	0.3	(0.8)
	Eating Habits (/16)	17	(71)	3.2	(3.5)	14	(64)	4.2	(5.1)
	Sleep (/8)	17	(71)	2.2	(2.0)	14	(64)	1.4	(1.2)
	Stereotypic and Motor Behaviours (/16)	17	(71)	4.2	(4.5)	14	(64)	5.3	(5.0)
	Motivation (/20)	17	(71)	7.4	(6.5)	14	(64)	8.9	(7.0)
	Total (/180)	17	(71)	55.5	(32.6)	14	(64)	51.2	(29.0)
Cognition (Z-scores)	Memory recall	20	(83)	−2.0	(1.1)	16	(73)	−1.3	(1.0)
	Delayed recognition memory	16	(67)	−1.6	(1.5)	10	(45)	−1.0	(1.2)
	Executive function	20	(83)	−1.3	(0.8)	19	(86)	−1.5	(0.7)
	Language	20	(83)	−1.3	(0.9)	18	(82)	−1.6	(1.3)
	Attention and processing speed	19	(79)	−1.2	(1.3)	19	(86)	−1.0	(1.0)
	Visuospatial function	17	(71)	−0.9	(1.2)	18	(82)	−0.4	(1.0)
Biomarker (pg/mL)	CSF NfL (HC: <1036 [908, 1165])^c^	24	(100)	911.0	(288.9)	22	(100)	1451.0	(1470.5)
	CSF Aβ42 (HC: >544 [434.0, 580.3])^d^	24	(100)	459.8	(186.0)	22	(100)	704.5	(231.8)
	CSF T-tau (HC: <407 [379.7, 513.8])^d^	24	(100)	507.5	(248.7)	22	(100)	252.3	(193.8)
	CSF P-tau181 (HC: <78 [70.4, 86.1])^d^	24	(100)	86.0	(30.7)	21	(95)	45.0	(24.0)

*Notes*: Aβ, amyloid-β; AD, Alzheimer’s disease; CBI-R, Cambridge Behavioural Inventory – Revised; CSF, cerebrospinal fluid; DASS-21, Depression Anxiety Stress Scale – 21; HC, healthy control; MCI, mild cognitive impairment; µg/mL, microgram per millilitre; NfL, neurofilament light chain protein; NPS, neuropsychiatric symptoms; N, sample size; P-tau181, phosphorylated tau 181; pg/mL, picogram per millilitre; SD, standard deviation; T-tau, total tau.

a Percentage within the YOAD subgroup (n = 24).

b Percentage within the non-AD dementias subgroup (n = 22).

c Normative reference range and 95% confidence interval for CSF NfL values derived from Eratne et al. (2020).

d Normative reference ranges and 95% confidence intervals for CSF Aβ42, T-tau, and P-tau181 derived from Li et al. (2015).

The mean (SD) age at symptom onset was 54.3 (7.6) years, and the mean (SD) age at diagnosis was 57.0 (7.5) years, with a mean (SD) illness duration of 2.4 (2.2) years. Participants had received a mean (SD) of 12.0 (2.5) years of education. Psychological distress, as measured by the DASS-21, had a mean (SD) total score of 19.1 (19.4). Behavioural and functional deficits, assessed via the CBI-R, had a mean (SD) total score of 53.6 (30.6).

Cognitive performance profiles varied across diagnostic subgroups. In YOAD, the most impaired domains were memory recall (mean [SD] Z-score = −2.0 [1.1]) and delayed recognition memory (Z = −1.6 [1.5]), followed by executive function (Z = −1.3 [0.8]), language (Z = −1.3 [0.9]), and attention and processing speed (Z = −1.2 [1.3]), with visuospatial function being the least affected (Z = −0.9 [1.2]). In non-AD dementias, the most affected domains were language (Z = −1.6 [1.3]) and executive function (Z = −1.5 [0.7]), followed by memory recall (Z = −1.3 [1.0]), delayed recognition memory (Z = −1.0 [1.2]), and attention and processing speed (Z = −1.0 [1.0]), with visuospatial function being relatively preserved (Z = −0.4 [1.0]).

### Associations Between Biomarkers and Cognition/NPS (Aim 1)

#### Biomarker-Cognition Associations

**Figure 1.**
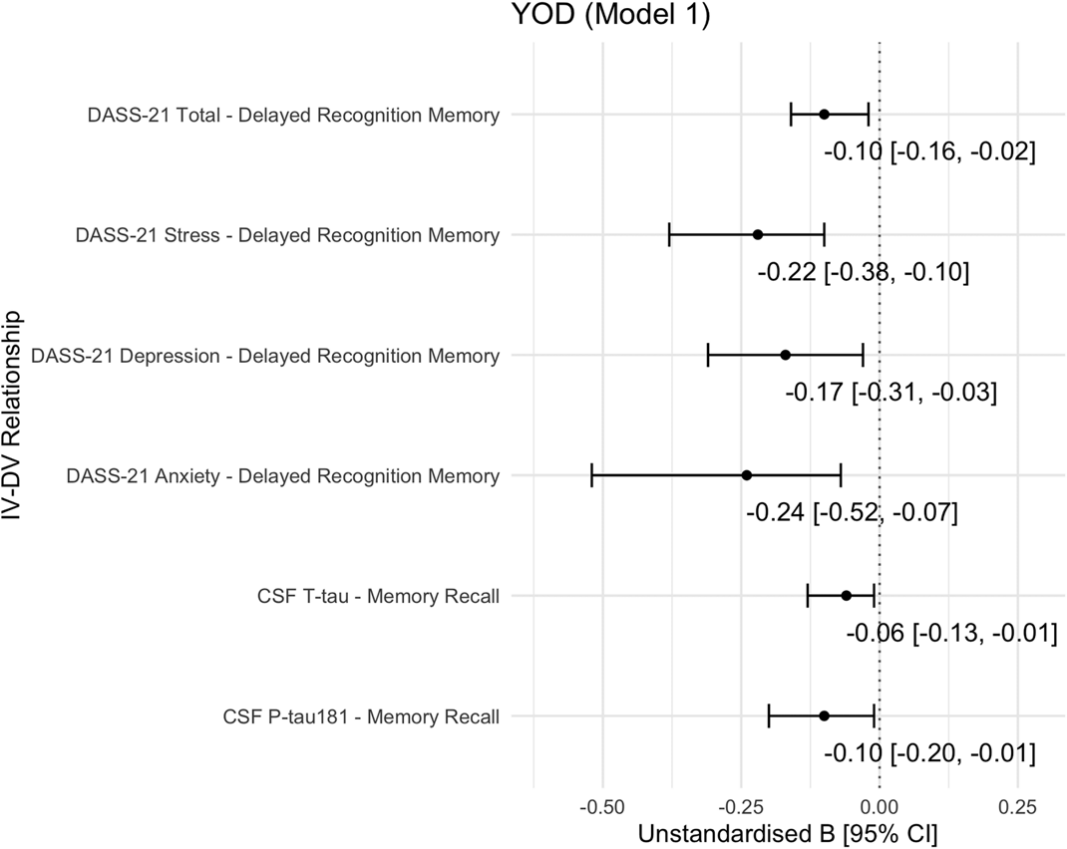
Summary of significant associations of biomarkers or neuropsychiatric symptoms with cognitive functions in the young-onset dementia cohort. *Note*: CI, confidence interval; CSF, cerebrospinal fluid; DASS-21, Depression Anxiety Stress Scale – 21; DV, dependent variable; IV, independent variable; P-tau181, phosphorylated tau 181; T-tau, total tau; YOD, young-onset dementia. Model 1 was adjusted for age, sex, and diagnosis. Models 2–5 are detailed in Supplementary Figure S1.

Among YOD participants (YOAD and non-AD dementias combined), memory recall was significantly associated with CSF P-tau181 and T-tau levels after adjustment for age, sex, and diagnosis. P-tau181 exhibited the strongest relationship (unstandardised beta [B] = −0.10, 95% CI = [−0.20, −0.01], AICc weight = 0.43), followed by T-tau (B = −0.06 [−0.13, −0.01], AICc weight = 0.48) (Figure 1). These associations remained significant after further adjustment for NfL levels (Supplementary Figure S1(a,b), Model 5). No significant biomarker-by-group interactions were observed, indicating consistent effects across the YOAD and non-AD dementias subgroups.

No significant relationships were observed between biomarkers and other cognitive domains.

#### Biomarker-NPS Associations

In the non-AD dementias subgroup, higher T-tau levels were associated with greater behavioural and functional deficits, as measured by total CBI-R scores (B = 0.76 [0.06, 3.52], AICc weight = 0.95) (Supplementary Figure S2, Model 1). This association was independent of age, sex, diagnosis, and NfL. No such associations were observed in the YOAD subgroup.

No significant associations between biomarkers and other neuropsychiatric dimensions were identified.

### Associations Between NPS and Cognition and Moderation/Mediation (Aim 2)

#### NPS-Cognition Associations

In participants with YOD, total DASS-21 scores significantly linked to delayed recognition memory performance (B = −0.10 [0.16, −0.02], AICc weight = 0.97) (Figure 1), independent of age, sex, and diagnosis. This association remained significant after further adjustment for NfL levels.

Sensitivity analyses of the DASS-21 subscales revealed that *Anxiety* (B = −0.24 [−0.52, −0.07]) and *Stress* (B = −0.22 [−0.38, −0.10]) had the strongest relationships with delayed recognition memory, followed by *Depression* (B = −0.17 [−0.31, −0.03]) (Figure 1). *Anxiety* and *Stress* remained as significant correlates after adjusting for each CSF biomarker separately (Aβ42, P-tau181, T-tau, and NfL), indicating biomarker-independent effects (Supplementary Figure S1(e,f), Model 2–5). In contrast, the association with *Depression* became insignificant after biomarker adjustment. No significant NPS-by-group interactions were found, suggesting consistent associations across the YOAD and non-AD dementias subgroups.

#### Moderation and Mediation

No evidence was found for CSF biomarkers acting as moderators or mediators in the observed NPS-cognition relationships.

## Discussion

This study examined the interrelationships between NPS, cognitive performance, and CSF biomarkers in a clinical cohort of YOD diagnosed with multiple neurodegenerative conditions. Three major findings emerged: (1) CSF P-tau181 and T-tau were significantly associated with memory recall across the YOD group, (2) T-tau and NfL showed distinct patterns of association with NPS and cognition, suggesting heterogeneous neurodegenerative mechanisms, and (3) anxiety and stress were associated with delayed recognition memory independently of all core biomarkers. Together, these results suggest potential mechanisms linking tau pathology, affective symptoms, and cognitive deficits in younger individuals with dementia.

Among participants with YOD, P-tau181 and T-tau levels were significantly associated with memory recall, independent of NfL, and consistent across both YOAD and non-AD dementias. These findings align with prior studies linking tau pathology to cognitive dysfunction, particularly in memory-related networks such as the hippocampus and medial temporal cortex (Bejanin *et al*., 2017; Mielke *et al*., 2017; Hanseeuw *et al*., 2019; Pase *et al*., 2019). Rather than simply reflecting a general disease burden, elevated tau may contribute to memory impairment through disruption of synaptic signalling and neuronal integrity in these regions. The lack of interaction with diagnostic subgroups suggests a shared tau-driven pathway to memory impairment across the YOD subtypes, supporting the hypothesis that tau accumulation contributes directly to memory decline across distinct clinical aetiologies (Biundo *et al*., 2018).

In contrast, NfL was not significantly associated with memory or other cognitive domains in this cohort. This diverges from previous studies reporting relationships between NfL and global cognitive scores in AD and Parkinson’s disease (Lin *et al*., 2018; Gu *et al*., 2023) and with executive function in YOD (Walia *et al*., 2022). However, these latter studies often employed brief cognitive screening tools (e.g., the Mini-Mental State Examination and the Neuropsychiatry Unit Cognitive Assessment Tool), which capture general impairment but lack sensitivity to domain-specific deficits. In the present study, formal neuropsychological assessments were used to quantify discrete cognitive domains, allowing finer-grained analyses. It is plausible that NfL, as a marker of diffuse axonal degeneration, relates more closely to overall cognitive efficiency than to specific processes such as memory encoding or retrieval. This may explain the discrepancy with prior studies and highlights the importance of using sensitive, domain-level measures when examining biomarker-cognition relationships.

T-tau was found to be significantly associated with NPS severity, specifically in individuals with non-AD dementias. This supports earlier findings implicating tau pathology in behavioural disturbances, particularly in FTD and other non-AD dementias, where fronto-limbic networks governing emotion regulation are disrupted (Bloniecki *et al*., 2014; Babulal *et al*., 2016; Ramakers et al. 2013; Skogseth *et al*., 2008). The strength of this association, independent of NfL, reinforces the role of T-tau as an index of cortical neuronal injury contributing to behavioural and emotional dysregulation. Given that elevated T-tau reflects active neuronal injury, particularly in early disease stages (Cotta Ramusino *et al*., Cotta Ramusino *et al*., 2021), this may represent a period in which tau-related cortical degeneration directly influences behavioural symptoms.

Conversely, NfL was not associated with NPS severity in our cohort. As a marker of large-calibre axonal degeneration, NfL is more indicative of white matter damage (Khalil *et al*., 2018), which may be less proximally involved in the expression of behavioural symptoms compared to cortical and limbic neuronal damage captured by T-tau (Zetterberg *et al*., Zetterberg *et al*., 2013). This pattern supports the idea that T-tau and NfL reflect complementary but anatomically distinct pathological processes, cortical versus subcortical degeneration, each contributing differently to the clinical phenotype (Dhiman *et al*., 2020; Fourier *et al*., 2020; Giuffrè et al. 2023; Marks et al. 2021).

Anxiety and stress symptoms were associated with impaired delayed recognition memory, even after adjusting for Aβ42, P-tau181, T-tau, and NfL. This finding indicates that affective dysregulation may contribute to cognitive vulnerability through non-degenerative biological pathways Chronic stress is known to affect the hypothalamic-pituitary-adrenal axis, leading to prolonged cortisol exposure, hippocampal atrophy, and impaired neurogenesis, all of which may adversely impact memory (Ismail *et al*., 2018). Additionally, neuroinflammatory activation and altered monoaminergic signalling may further exacerbate these effects (Hassamal, 2023).

In contrast, depression may reflect a different clinical process. While anxiety and stress may represent ‘reactive’ responses to emerging cognitive difficulties, depression in YOD may be ‘pathological’, arising from neurodegenerative changes within limbic and fronto-subcortical regions (Stella *et al*., 2014; Ismail *et al*., 2018). Clinically, distinguishing reactive affective symptoms from those driven by underlying neuropathology is critical for diagnostic accuracy and for tailoring treatment and caregiver interventions.

Contrary to prior studies reporting that biomarkers may moderate and mediate the relationship between NPS and cognition across the AD continuum (Gatchel et al. 2019; Johansson *et al*., 2020; Ng *et al*., 2021; Xu *et al*., 2024), no such effects were detected in this cohort. Several factors may account for this discrepancy. First, the relatively small sample size may have limited the statistical power to detect moderation or mediation effects, which typically require larger samples for adequate detection. Second, the heterogeneity of our YOD cohort, comprising YOAD, FTD, VaD, and other neurodegenerative conditions, likely introduced variability in neuropathological substrates that influence the relationship between NPS and cognition differently across diseases. This diagnostic diversity may have diluted effect sizes and contributed to the absence of biomarker moderation or mediation effects.

This study’s strengths lie in its focus on the under-researched population of YOD and its comprehensive integration of cognitive, neuropsychiatric, and biomarker data. However, several limitations must be considered. The cross-sectional design precludes causal inferences and offers only a single time-point assessment of biomarker-symptom-cognition relationships, limiting insights into their temporal progression. A longitudinal approach is essential to understanding how biomarkers evolve and predict symptom and cognitive trajectories over time. Additionally, as CSF samples were collected and stored over more than a decade, prolonged storage may have affected biomarker stability. Although Aβ42, tau, and NfL were measured using validated and quality-controlled assays, the potential impact of long-term storage on analyte integrity cannot be fully excluded and may have introduced minor variability across samples collected during the 12-year study period.

The relatively small sample size reduces statistical power and limits generalisability, particularly when comparing individual YOD subtypes such as FTD and VaD. Larger, multi-centre cohorts with well-characterised diagnostic subtypes and longitudinal follow-up would improve statistical robustness and enhance the clinical applicability of findings. Expanding biomarker analyses to include advanced imaging, neuroinflammatory markers, and proteomic studies could provide a more comprehensive understanding of the complex interplay between NPS, such as anxiety and stress, and cognitive impairment in younger individuals with dementia.

Another important consideration is the issue of multiple comparisons, which increases the risk of Type I error. While FDR correction was applied, none of the adjusted *p*-values were significant at *p* < 0.05 (see Supplementary Table S2). Given the exploratory nature of this study, uncorrected results were reported to maximise sensitivity and identify potential associations for future research. However, these findings require cautious interpretation (as hypothesis-generating), and validation in larger, independent cohorts using stringent statistical approaches is necessary to ensure the reproducibility of results. Future studies should prioritise robust analytical methods that accounts for multiple comparisons while maintaining adequate statistical power to detect meaningful associations.

## Conclusion

This study contributes to understanding how CSF biomarkers relate to neuropsychiatric and cognitive features in YOD. Findings suggest that tau-mediated neurodegeneration, reflected by P-tau181 and T-tau, may be more closely associated with memory impairment than axonal injury markers such as NfL. The association between T-tau and NPS in non-AD dementias highlights its potential role in behavioural symptomatology, while the lack of a relationship between NfL and NPS suggests distinct neurodegenerative processes. Future research should focus on validating these associations in larger, longitudinal cohorts and refining biomarker-based diagnostic models. Incorporating multimodal approaches, including imaging and neuroinflammatory markers, will further elucidate the complex interplay between pathology, cognition, and symptomatology in YOD.

## Supporting information

Chiu et al. supplementary material Chiu et al. supplementary material

## Data Availability

The corresponding author has full access to all the data in the study and can share upon reasonable request.

## Supplementary material

The supplementary material for this article can be found at https://doi.org/10.1017/neu.2025.10051.
