# Supplementary material for: Distinct contributions of cerebrospinal fluid biomarkers to cognitive impairment and neuropsychiatric symptoms in young-onset dementia
Source: Acta Neuropsychiatr. 2025 Dec 23;38:e8. doi: 10.1017/neu.2025.10051 (PMC13130305; doi:10.1017/neu.2025.10051)
Supplement: Chiu et al. supplementary material [file S0924270825100513sup001.docx]

# Supplementary Materials

## Table S1. Summary of neuropsychological tests categorised into cognitive domains according to the primary domain underpinning the test.

| Cognitive domain | Neuropsychological test |
| --- | --- |
| Memory recall | - WMS-IV: Logical memory II (Wechsler 2009) - HVLT-R: Long delay task (Hester et al. 2004) - CVLT-L/S: Long delay free recall (Woods et al. 2006) - RCFT: 30-minute delay recall (Meyers and Meyers 1995; Strauss et al. 2006) |
| Delayed recognition memory | - CVLT-L/S: Recognition (Woods et al. 2006) - HVLT-R: Discrimination index (Hester et al. 2004) |
| Executive function | - DKEFS-VF: Letter fluency (Delis et al. 2001) - DKEFS-VF: Category switching (Delis et al. 2001) - DKEFS-CWI: Inhibition (Delis et al. 2001) - DKEFS-TMT: 4 Number-letter (Delis et al. 2001) - TMT B (Tombaugh 2004) - Hayling & Brixton Tests: Total score (Burgess and Shallice 1997) - WAIS-IV: DSB (Wechsler 2008) |
| Language | - DKEFS-VF: Category fluency (Delis et al. 2001) - Category fluency (animals only) (Tombaugh et al. 1999) - BNT: Total score (Tombaugh and Hubiey 1997) |
| Attention and processing speed | - WAIS-IV: Coding (Wechsler 2008) - WAIS-IV: Symbol search (Wechsler 2008) - DKEFS-CWI: Colour naming (Delis et al. 2001) - DKEFS-CWI: Word reading (Delis et al. 2001) - DKEFS-TMT: 1 Visual scan (Delis et al. 2001) - DKEFS-TMT: 2 Number (Delis et al. 2001) - DKEFS-TMT: 3 Letter (Delis et al. 2001) - DKEFS-TMT: 5 Motor speed (Delis et al. 2001) - TMT A (Tombaugh 2004) - WAIS-IV: Digit span (Wechsler 2008) |
| Visuospatial function | - RCFT: Copy (Meyers and Meyers 1995; Strauss et al. 2006) - WAIS-IV: Block design (Wechsler 2008) |
| **Abbreviations**: BNT, Boston Naming Test; CVLT-L/S, California Verbal Learning Test – Long/Short Form; CWI, Colour Word Interference; DKEFS, Delis–Kaplan Executive Function System; HVLT-R, Hopkins Verbal Learning Test – Revised; RCFT, Rey Complex Figure Test; TMT, Trial Making Test; VF, Verbal Fluency; WAIS-IV, Wechsler Adult Intelligence Scale – Fourth Edition; WMS-IV, Wechsler Memory Scale – Fourth Edition. | |

## Table S2. Summary of associations in the total cohort and subgroups, with correction for multiple comparisons.

| Predictor | Outcome | GLM  B [95% CI] | *p*-value  (FDR-corrected) |
| --- | --- | --- | --- |
| **YOD** |  |  |  |
| CSF P-tau181 | Memory recall | -0.10 *  [-0.20, -0.01] | 0.108 |
| CSF T-tau | Memory recall | -0.06 *  [-0.13, -0.01] | 0.108 |
| DASS-21 Total | Delayed recognition memory | -0.10 *  [-0.16, -0.02] | 0.090 |
| DASS-21 Depression | Delayed recognition memory | -0.17 *  [-0.31, -0.03] | 0.108 |
| DASS-21 Anxiety | Delayed recognition memory | -0.24 *  [-0.52, -0.07] | 0.090 |
| DASS-21 Stress | Delayed recognition memory | -0.22 **  [-0.38, -0.10] | 0.090 |
| **Non-AD dementias** |  |  |  |
| CSF T-tau | CBI-R Total | 0.76 *  [0.06, 3.52] | 0.112 |
| **Abbreviations**: Aβ, amyloid-β; B, unstandardised regression coefficient; CBI-R, Cambridge Behavioural Inventory – Revised; CI, confidence interval; CSF, cerebrospinal fluid; DASS-21, Depression Anxiety Stress Scale – 21; FDR, false discovery rate; GLM, general linear model; NfL, neurofilament light chain protein; P-tau181, phosphorylated tau 181; T-tau, total tau; YOD, young-onset dementia; YOAD, young-onset Alzheimer’s disease.  **Footnotes**:  General linear models (GLMs) assessed associations of neuropsychiatric symptoms and biomarkers (independent variable; predictor) with cognitive performance (dependent variable; outcome). Covariates included age, sex, and diagnosis. Correction for the false discovery rate was applied to the GLMs to account for multiple comparisons.  * p < 0.05 ** p < 0.01 *** p < 0.001 | | | |

## Figure S1. Summary of significant associations of biomarkers or neuropsychiatric symptoms with cognitive functions in the young-onset dementia cohort.


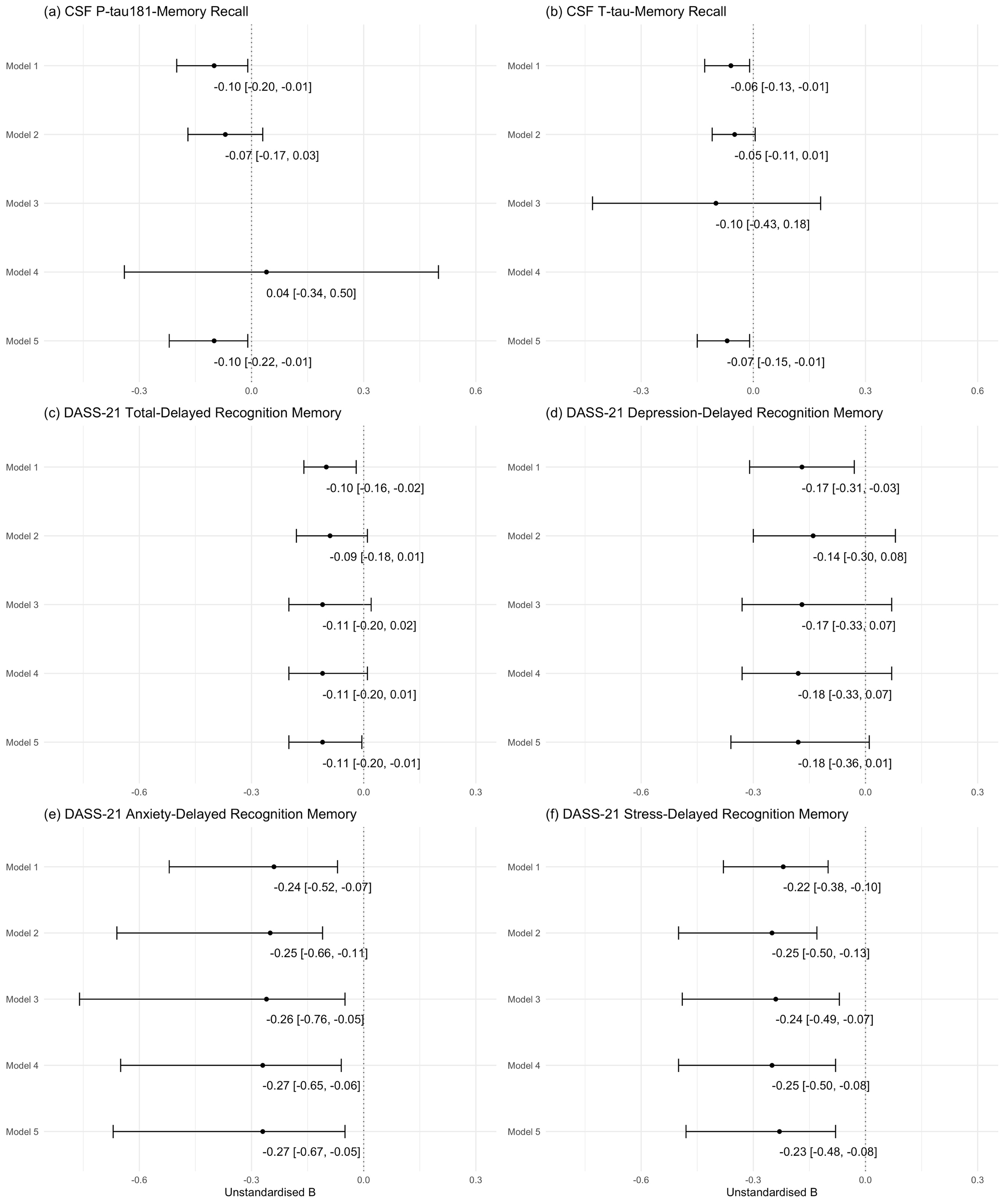


**Abbreviations**: CSF, cerebrospinal fluid; DASS-21, Depression Anxiety Stress Scale – 21; NfL, neurofilament light chain protein; P-tau181, phosphorylated tau 181; T-tau, total tau.

**Footnotes**:

Model 1 was adjusted for age, sex, and diagnosis.

Model 2 was adjusted for age, sex, diagnosis, and CSF Aβ42 levels.

Model 3 was adjusted for age, sex, diagnosis, and CSF P-tau181 levels.

Model 4 was adjusted for age, sex, diagnosis, and CSF T-tau levels.

Model 5 was adjusted for age, sex, diagnosis, and CSF NfL levels.

## Figure S2. The association between CSF T-tau and behavioural symptoms in non-AD dementias across different generalised linear models.


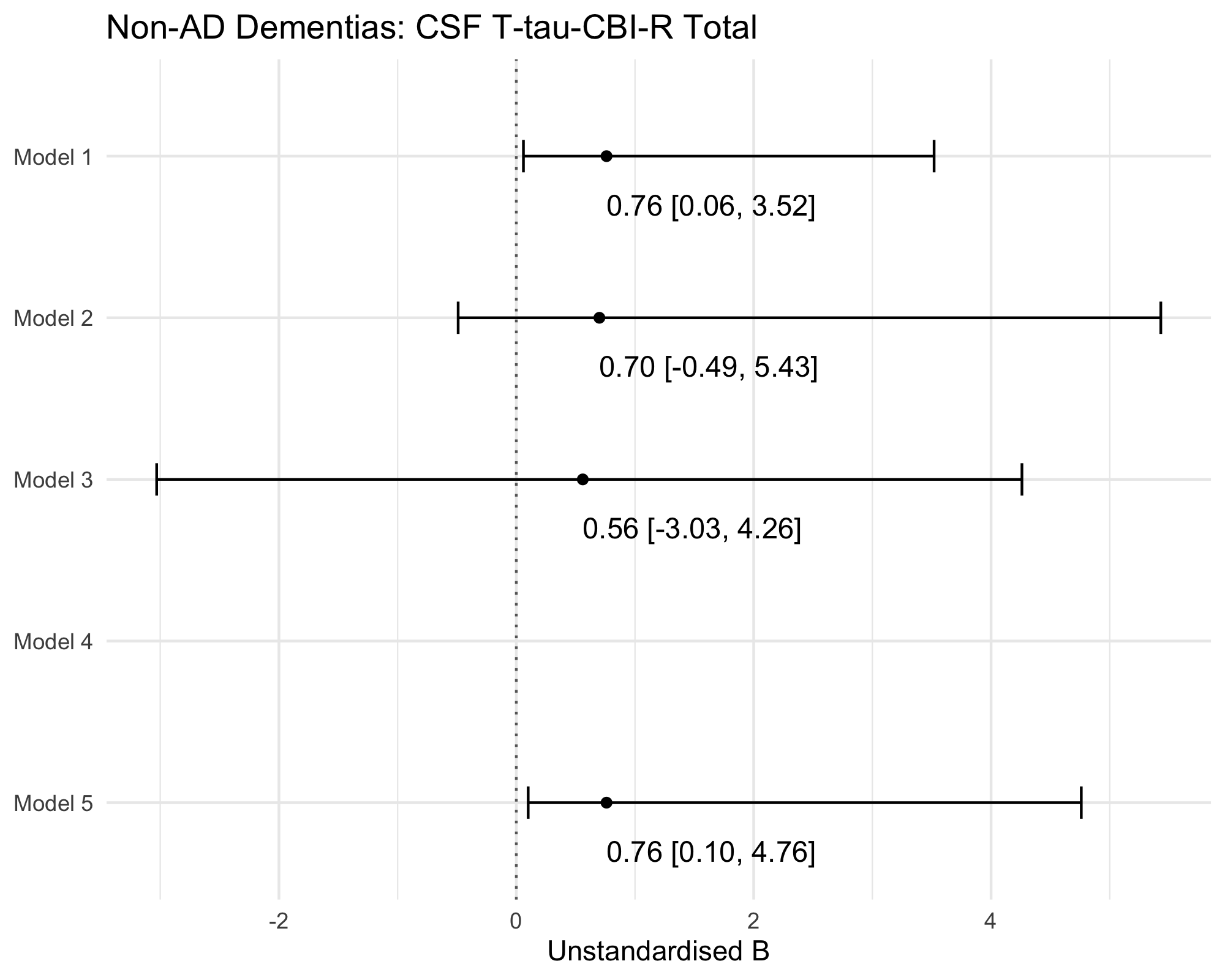


**Abbreviations**: CBI-R, Cambridge Behavioural Inventory – Revised; CSF, cerebrospinal fluid; NfL, neurofilament light chain protein; T-tau, total tau; non-AD dementias, non-Alzheimer’s disease dementias.

**Footnotes**:

Model 1 was adjusted for age, sex, and diagnosis.

Model 2 was adjusted for age, sex, diagnosis, and CSF Aβ42 levels.

Model 3 was adjusted for age, sex, diagnosis, and CSF P-tau181 levels.

Model 4 was adjusted for age, sex, diagnosis, and CSF T-tau levels.

Model 5 was adjusted for age, sex, diagnosis, and CSF NfL levels.
